# Prevalence of heteroresistant Helicobacter pylori and treatment follow-up in patients in Ilam, Iran

**DOI:** 10.3205/dgkh000479

**Published:** 2024-05-17

**Authors:** Sanaz Hosseini, Nahid Mahdian, Leila Gheitani, Mina Mahmoudi, Mohammad Raftari, Ali Hematian, Parvin Bahmaninejad, Behzad Badakhsh, Sobhan Ghafourian

**Affiliations:** 1 Department of Gastroenterology, Faculty of Medicine, Ilam University of Medical Sciences, Ilam, Iran; 2 Department of Microbiology, Faculty of Medicine, Ilam University of Medical Sciences, Ilam, Iran; 3 Clinical Microbiology Research Center, Ilam University of Medical Sciences, Ilam, Iran

**Keywords:** heteroresistance, Helicobacter pylori, recurrent infection, targeted antibiotic therapy, levofloxacin, follow-up

## Abstract

**Background:**

Special antibiotics are prescribed against *Helicobacter (H.) pylori*. However, sometimes the bacteria are not completely eliminated, or they are recurrent. Unlike most infections, it is very difficult to eliminate a *H. pylori* infection. Heteroresistance is defined as the phenomenon in which subpopulations of the same colony of bacteria exhibit a range of susceptibilities to a particular antibiotic. Because of heteroresistant cells, antibiotic failure and chronic infection can occur; thus, the current research aimed to investigate presence of heteroresistant cells in *H. pylori* collected from patients reffering to clinic in Ilam, Iran. Subsequently, patients who were infected with heteroresistant *H. pylori* were treated with antibiotics effective against heteroresistant subpopulations.

**Methods:**

In this cross-sectional descriptive study, 100 patients with clinical symptoms and suspected of being infected with *H. pylori* were studied in private clinics in Ilam, Iran. Fiftyisolates of *H. pylori* accompanied by patients’ information were obtained from Ilam clinics. We cultured the bacteria to identify heteroresistance and to find the cause of recurrent infection in these patients.

**Results:**

Out of a total of 50 samples, 3 were heteroresistant to clarithromycin (6%). Levofloxacin was applied in cases of heteroresistant samples, and the effectiveness was determined after one month of follow-up of patients.

**Conclusion:**

Patients with heteroresistance showed sensitivity to levofloxacin. After one month of follow-up, it was found that the effectiveness of this antibiotic was good. Therefore, this antibiotic was introduced as a more effective drug in patients with heteroresistant *H. pylori*.

## Abstract (de)

Zur Eradikation von *Helicobacter (H.) pylori* werden spezielle Antibiotika verschrieben. Manchmal werden nicht alle Bakterien abgetötet oder sie treten wieder auf. Anders als bei den meisten Infektionen ist es sehr schwierig, eine *H. pylori*-Infektion zu beseitigen. Heteroresistenz ist definiert als ein Phänomen, bei dem Subpopulationen derselben Species eine unterschiedliche Empfindlichkeit gegenüber einem bestimmten Antibiotikum aufweisen. Aufgrund der Heteroresistenz kann es zum Versagen des Antibiotikums und zu einer chronischen Infektion kommen. Daher sollte das Vorhandensein heteroresistenter Zellen von Patienten aus Ilam, Iran, von *H. pylori* untersucht werden. Die Patienten mit Vorkommen heteroresistenter *H. pylori* Subpopulationen wurden mit gegen diese Erreger wirksamen Antibiotika behandelt.

## Introduction

*Helicobacter pylori* is a rod-shaped Gram-negative non-spore-forming bacterium, has 4–6 polar filaments and grows under microaerophilic conditions [1]. The optimum temperature for its growth is 35–37°C. High humidity increases its growth. Most strains require 3–5 days and sometimes 7 days of incubation [2].

This bacterium is found in large numbers mainly on the mucosal surface of the stomach of carriers and can continue to grow by penetrating the sub-mucinous layer. *Helicobacter pylori* is able to laterally infect the gastric mucosa for decades, despite an acquired and inflammatory immune response as well as continuous gastric epithelial replacement [3], [4]. Poor hygiene, inadequate water supply, and overcrowding living conditions are among the factors that increase the prevalence [5].

*Helicobacter pylori* is the most common microscopic organism that infects humans worldwide [6]. More than half of the world’s population is infected with this bacterium. It is considered the main cause of diseases such as stomach ulcers and intestinal disorders [7]. It is also the main cause of stomach cancer; accordingly, 90% of people with stomach cancer are infected with *H. pylori*. However, *H. pylori* infection alone is not enough to cause stomach cancer; the presence of other risk factors is necessary [4].

Special antibiotics are prescribed against *Helicobacter (H.) pylori*, but they do not always completely eradicate the bacteria, and colonies may become reestablished. Unlike most infections, it is very difficult to eliminate a *H. pylori* infection, because this bacterium lives safely in the stomach mucosa and protects itself from medication. Simultaneous treatment with four drugs for a period of two weeks is necessary for the successful eradication of *H. pylori*. In dealing with infections involving of this microbe, it is of paramount importance to complete the treatment period to ensure success.

Sometimes the reason for failed antimicrobial therapy against *H. pylori* is antibiotic resistance; recently, Bahmaninejad et al. [8] reported that resistance may be caused by persister cells (subpopulations of cells that resist treatment, and become tolerant to antimicrobials by changing to a state of dormancy or quiescence). Another possible reason for antimicrobial failure could be heteroresistance. Identifying heteroresistance in sensitive or resistant groups is often difficult. Finally, they may be reported as sensitive groups, and this may cause recurrence of infection or treatment failure [9] because it may be heteroresistance. To deal with this problem, not only new treatment strategies are needed, but also a better understanding of the emergence and spread of drug-resistant bacteria. In addition, better diagnostic methods are required to guide physicians in choosing optimal antimicrobial treatment methods. Over the past decades, our understanding of the mechanism of antibiotic resistance has greatly increased. The presence of resistance mechanisms (including mutations and resistance genes) in the bacterial cell can lead to phenotypic resistance. However, there are exceptions. Apparently identical bacterial cells in a population can show phenotypic heterogeneity in terms of antibiotic sensitivity, which leads to problems in classifying bacteria as sensitive or resistant [10].

Heteroresistance is a bacterial phenotype in which a bacterial isolate contains subpopulations with higher antibiotic resistance compared to the main population. This resistant subpopulation is often difficult to detect and poses a clinical concern, as it may increase upon repeated antibiotic exposure and cause treatment failure. Although this phenotype of bacteria was reported for the first time in 1940, their study was long delayed due to the lack of standard identification methods. Today, in addition to the availability of standard identification methods for this phenotype, research on this phenotype is intensifying in order to determine its etiology [10].

Because of heteroresistant cells, antibiotic failure chronic infection may occur. Thus, the current research aimed to investigate presence of heteroresistant cells in *H. pylori* collected from patients from Ilam, Iran. Patients who were infected with heteroresistant *H. pylori* were treated with anti-heteroresistance antibiotics.

## Methods

### Bacterial collection and identification

*H. pylori* was previously collected and identified by Bhamaninejad et al. [9], who provided us with samples for analysis of heteroresistance, enabling us to following the suspected patients.

### Antibiotic susceptibility testing (AST)

A broth micro dilution method based on Clinical and Laboratory Standards Institute (CLSI) was used to measure the sensitivity to clarithromycin, metronidazole and tetracycline. The minimum inhibitory concentrations (MIC) were calculated for all antibiotics.

### Determination of heteroresistant H. pylori

The standard population analysis profiles (PAP) method was used to determine heteroresistant *H. pylori*. Analysis was performed among *H. pylori* isolates which were sensitive to antibiotics. *E. coli Top10* was used as the quality control organism. Briefly, the PAP method includes: serial dilutions of a full 24-h culture (adjusted to 10^8^ colony-forming units [cfu/ml]), ranging from 10^8^–10^2^ cfu/ml in CAMHB were prepared. 50 ml of each dilution was cultured on Brucella blood agar plates containing 0.5, 1, 2, 3, 4, 5 and 6 times the MIC concentration for each antibiotic individually. After 24 hours of incubation at 37°C, the colonies on each plate were counted.

Colonies of heteroresistant *H. pylori*, is defined as a subpopulation of antibiotic-sensitive isolates that which can grow at almost 4 times the MIC, were selected and sub-cultured in antibiotic-free LB broth for the subsequent experiments. The frequency of heteroresistant subpopulations was calculated by dividing the number of colonies that grew on the plate with the maximum concentration of antibiotic by the colony counts from the plate without antibiotics.

### Follow-up of the patients

Finally, the patients were evaluated for one month after taking antibiotics to determine the recurrence of infection and treatment. Stool samples were taken from the patients and the amount of *H. pylori* antigen was checked using the antigen *H.pylori* detection ELISA kit.

## Results

### Presence of heteroresistance in H. pylori isolates

Of 50 *H. pylori* isolates, 3 isolates were heteroresistant to clarithromycin (6%). The PAP method was used to determine whether the samples contained heteroresistant cells. *E. coli *Top10, which does not have the ability to form heteroresistance, was used as the negative control. Finally, using this method, among the samples that were phenotypically sensitive to clarithromycin and tetracycline, 3 grew on Brucella blood agar medium, and they were reported as heteroresistant.

### Elimination of heteroresistance with levofloxacin

In the next step, levofloxacin was applied to heteroresistance samples, and the effectiveness of this antibiotic in patients was determined after one month of follow-up. Clinical symptoms in patients with heteroresistance were resolved with levofloxacin administration, demonstrating the efficacy of this antibiotic.

## Discussion

After identifying the heteroresistance samples, 3 of the 50 samples were resistant to clarithromycin (6%). After performing heteroresistance testing using the PAP method among samples that were phenotypically sensitive to clarithromycin, 3 of them grew on Brucella blood agar medium and heteroresistance was reported.

In the next step, the antibiotic levofloxacin was tested in heteroresistance samples, and the effectiveness of this antibiotic was determined in patients after one month of follow-up, with levofloxacin showing greater efficacy. in the patients who had heteroresistance. Their clinical symptoms were resolved.

In the study by Kocsmar et al. [11], the total resistance rate was 23.9% (73 cases), in which 38 isolates (12.5%) were heteroresistant. Our study demonstrated that 6% of *H. pylori* were heteroresistant and showing antimicrobial failure after initial treatment but after administration of levofloxacin, heteroresistant *H. pylori* was eradicated. 

## Conclusion

The current study reports the first successful therapy in patients with heteroresistant *H. pylori*. The study was limited by the low number of small sample size, so that we cannot definitively state that levofloxacin is an effective antibiotic against heteroresistant *H. pylori*. Also, it should be noted that levofloxacin was effective against persister cells in the sample from Bahmaninejad et al. [8]. We highly recommended that this research be continued in a larger population and a different geographical area.

## Notes

### Competing interests

The authors declare that they have no competing interests.

### Authors’ ORCID

- Sobhan Ghafourian: 0000-0002-2142-1600
- Behzad Badakhsh: 0000-0002-3112-2129
